# Endometrial thickness following early miscarriage in IVF patients – is there a preferred management approach?

**DOI:** 10.1186/s12958-021-00780-7

**Published:** 2021-06-22

**Authors:** Gilad Karavani, Heli Alexandroni, Daniel Sheinin, Uri P. Dior, Alex Simon, Assaf Ben-Meir, Benjamin Reubinoff

**Affiliations:** 1 Infertility and IVF Unit, Department of Obstetrics and Gynecology, Hadassah Ein-Kerem Medical Center and Faculty of Medicine, Hebrew University of Jerusalem, Jerusalem, Israel; 2 Department of Obstetrics and Gynecology, Shaare Zedek Medical Center and Faculty of Medicine, Hebrew University of Jerusalem, Jerusalem, Israel; 3 Faculty of Medicine, Hebrew University of Jerusalem, Jerusalem, Israel

**Keywords:** Endometrial thickness, Miscarriage, Expectant management, Misoprostol, Dilation and curettage

## Abstract

**Background:**

Endometrial thickness (ET) has previously been shown to positively correlate with implantation and clinical pregnancy rates. Pregnancies achieved using in-vitro fertilization (IVF) technique are prone to higher rates of early miscarriage. The aim of this study was to compare the effects of expectant management, medical treatment (Misoprostol) and dilation and curettage (D&C) for early miscarriage following IVF cycles on the subsequent cycle outcomes - endometrial thickness and reproductive outcomes.

**Methods:**

A retrospective cohort study of women who underwent embryo transfer, conceived and had first trimester miscarriage with at least one subsequent embryo transfer. ET measurements during fresh or frozen-thawed IVF cycles were assessed for each patient. Comparisons of ET differences between the miscarriage and the subsequent cycles, as well as reproductive outcomes, were performed according to the initial miscarriage management approach.

**Results:**

A total of 223 women were included in the study. Seventy-eight women were managed conservatively, 61 were treated with Misoprostol and 84 women underwent D&C. Management by D&C, compared to conservative management and Misoprostol treatment was associated with higher prevalence of a significant (> 2 mm) ET decrease (29.8%% vs. 14.1and 6.6%, respectively; *p* < .001) and was the only approach associated with a significant increase in the rates of ET under 7 and 8 mm in the following cycle (*p* = 0.006 and 0.035; respectively).

Clinical pregnancy rates were significantly lower following D&C compared with conservative management and Misoprostol (16.7% vs. 38.5 and 27.9%, respectively; *p* = 0.008) as well as implantation rate (11.1% vs. 30.5.% and 17.7, respectively; *p* < 0.001).

**Conclusion:**

Our data suggest that D&C management of a miscarriage is associated with decreased ET and higher rates of thin endometrium in the subsequent IVF cycle, compared with conservative management and Misoprostol treatment. In addition, implantation and pregnancy rates were significantly lower after D&C.

**Supplementary Information:**

The online version contains supplementary material available at 10.1186/s12958-021-00780-7.

## Introduction

The success rate of an In-vitro fertilization (IVF) treatment depends on multiple factors. Among them are patient’s age, number and quality of transferred embryos, type of stimulation protocol and endometrial characteristics [1]. Features of the endometrium, evaluated with vaginal ultrasound (US), include endometrial thickness (ET), pattern, volume and blood flow [2].

The correlation between ET and implantation rate has been widely studied in the literature with inconsistent results. It is still uncertain whether there is a minimal ET threshold that is essential for successful implantation. Some studies concluded that a thin endometrium less than 7 mm (mm) is associated with a greater risk for unfavorable outcome of IVF treatments [3–9]. Others reported a decreased fecundity rate for endometrium thickness below 8–10 mm [10–14]. A thick endometrium has also been suggested to be associated with implantation failure. Weissman et al., reported reduced implantation and pregnancy rates in a group of patients with ET above 14 mm, compared to 7–14 mm [15]. Nonetheless, case reports showed that IVF pregnancies can be carried out even with a minimal ET of 4 mm [16] or an increased thickness, up to 20 mm [17]. Regardless of the inconclusive literature regarding the optimal ET, most studies agree that within the low range of ET there is a positive correlation between ET and implantation rate, particularly with pregnancies achieved by assisted reproductive technology (ART) [1, 15].

Pregnancies that are conceived via IVF are suggested to carry a slightly increased risk of miscarriage, compared with spontaneous conceptions [18]. Management of miscarriage include either expectant management, medical treatment (antiprogesterone or most commonly a prostaglandin analogue) or surgical uterine curettage known as dilation and curettage (D&C).

Due to a paucity of evidence regarding the effect of miscarriage management approach on subsequent ET and pregnancy outcomes, the dilemma which management approach is preferred remains unsolved. In this study, we sought to evaluate the effects of the different miscarriage management approaches on ET, clinical pregnancy and delivery rates in the subsequent IVF cycle. This study aims to provide data that may assist physicians when counseling patients with early miscarriage regarding the preferred management option.

## Materials and methods

### Study population

This retrospective cohort study included women that presented with an early miscarriage (first trimester) following a fresh or frozen-thawed (FT) embryo transfer in IVF cycles between 2006 and 2017.

Diagnosis of early miscarriage was made by vaginal US scan during a routine first trimester examination or following symptoms of abdominal contractions or vaginal bleeding. Patients with a miscarriage under 12 weeks (calculated according to the embryo transfer date) were included in the study. According to the management policy in our institution, each patient was fully informed and consulted about three miscarriage management possibilities: expectant management, medical treatment (Misoprostol) and dilation and curettage (D&C) followed by a specific consent form given and signed by the patients who chose medical or surgical approaches according to individual preferences.

The patients were divided into three management groups that were defined according to the initial miscarriage management approach that was selected by the patients.

The first group included patients who preferred expectant management and had undergone complete abortion by the time of follow-up visit, 2 weeks after the initial diagnosis. In cases of lack or incomplete spontaneous expulsion of pregnancy products from the uterine cavity within 2 weeks, medical or surgical interventions were performed according to patients’ choice. Such cases were not included in the study.

The second group included patients who chose medical treatment management. They received four 200mcg Misoprostol tablets (800mcg in total), inserted into the posterior fornix of the vagina. After 48-h, a second dose of 800mcg vaginal Misoprostol was given if US scan demonstrated a remaining gestational sac or thick endometrium (above 25 mm). Two weeks after the administration of Misoprostol, treatment was considered successful if an empty uterine cavity was demonstrated by a transvaginal US. Patients with successful medical management were included in the second group, while patients with retained products of conception who underwent further evacuation by D&C were not included in the study.

The third group included patients who preferred surgical management. They underwent, under general anesthesia, dilation of the cervix, vacuum aspiration of gestational products, followed by sharp curettage (D&C) to complete evacuation and confirm that the uterine cavity was empty. D&Cs were routinely performed by an attending physician, besides for cases that required an urgent procedure during on calls. In these cases, the D&Cs were performed by residents. Guidance of curettage by US was not routinely performed.

Exclusion criteria were miscarriages before 6 or beyond 12 weeks of gestation, chemical pregnancies (no evidence of gestational sac) and pregnancies suspected to be ectopic. Patients which experienced complications of miscarriage managements including excessive uterine bleeding (resulting in a need for blood transfusion), perforation of the uterus, fever and a need for operative hysteroscopy for the removal of retained products of conception were excluded. As mentioned above, patients with failed conservative or Misoprostol treatments that eventually underwent D&C procedure and for whom the initial and the final management approaches were different, were not included in the study to prevent a selection bias that may result in inferiority of the D&C group’s outcomes.

General and gynecological medical history was collected for all the patients. For each patient, at least two IVF cycles within 18 months were evaluated: the miscarriage cycle and the subsequent cycle. When the type of embryo transfer (fresh or frozen-thawed (FT)) in the miscarriage and subsequent cycles was different, the first additional subsequent cycle with a similar type of embryo transfer as the miscarriage cycle was also evaluated.

### Endometrial thickness evaluation

During each IVF cycle, ET was routinely measured when patients underwent US examination. We collected data regarding measurements at three points of each IVF cycle: at day three of menstrual period, at the day of hCG administration (fresh IVF cycles) or LH surge (natural frozen-thawed cycles) or first day of progesterone administration (estrogen and progesterone frozen-thawed cycles) (hCG/LH/P), and at “mid-cycle” between the 7th and 10th day of menstrual period). ET was defined as the maximal distance between the echogenic interfaces of the myometrium and the endometrium in a midsagittal plane of the uterus. Ultrasound examinations at day three were performed either at the community services or at our IVF unit. All ultrasound measurements at hCG/LH/P administration were performed at our IVF unit by a single US specialist in the same setting. These measurements were done by transvaginal US using a Voluson™ GE E6 system device and IC 5–9-D MHz transvaginal probe (GE Healthcare, Milwaukee, WI).

### IVF protocols and embryo transfer

Controlled ovarian stimulation included Gonadotropin-releasing hormone (GnRH) using either long or short agonist protocols or an antagonist protocol. Oocyte retrieval was performed under transvaginal ultrasound guidance and general anesthesia. Intracytoplasmic sperm injection (ICSI) was used in all cases of male factor infertility and in cases of unexplained infertility with previous low fertilization rates. Embryo quality was determined by cell number, symmetry and fragmentation and was graded “A-C”. Embryos were cultured in a one-step medium (“SAGE 1-step” (SAGE, Al-rad medical, Nes Ziona, Israel) at 37 °C, 5.7% CO2, and 5% O2 (Unisense FertiliTech, Aarhus, Denmark). At the time period of the study, transfer of embryos at cleavage stage was practiced in vast majority of cases (97%) in our unit. The number of embryos that were transferred was 1–3 embryos, in line with national fertility society guidelines. Two catheters were optional for embryo transfer (upon operator preference) - the Edwards-Wallace catheter (Classic Embryo Replacement Catheter; Smiths Medical, Hythe, Kent, U.K.) or the SIVF catheter (K-Jets-7019-SIVF; Cook IVF, Eight Miles Plains, Queensland, Australia). Frozen thawed embryo transfers were performed in natural cycles or artificial cycles using exogenous estradiol (2 mg Q8h) and vaginal progesterone supplementation (Utrogestan; Besins healthcare, Paris, France 400 mg Q12h).

### Outcomes

The primary outcome was alterations in ET following the three management approaches of miscarriage. ET assessed at the day of hCG/ LH/P during the primary miscarriage cycle was compared to both the subsequent and the first subsequent cycles with the same type of embryo transfer (fresh or thawed). This parameter was evaluated as a continuous as well as categorical (ET decrease of more than 2 mm and ET thinner than 6, 7 and 8 mm) parameter. Secondary outcomes included implantation, clinical pregnancy, miscarriage and live birth rates in the cycles that followed the miscarriage, comparing the three management approaches. Additional parameters assessed were infertility cause, type of IVF cycle and protocol, the baseline FSH (mIU/ml), the peak estradiol level (pmol/l), the total FSH given and the ET threshold for implantation. In fresh embryo transfer cycles, data regarding number of aspirated and mature oocytes, grade of embryos, quality and number of embryos transferred or frozen were also evaluated.

### Ethical approval

This study was approved by the Human Investigation Review Board of Hadassah Hebrew University Medical center (IRB 0657–17-HMO) and conforms to the provisions of the declaration of Helsinki.

### Statistical analysis

The association between categorical variables was tested using the Pearson χ2 test, as well as the Fisher’s exact test, as indicated. For quantitative variables, the comparison between independent variables of the three study groups in both the miscarriage cycle and the first cycle following the miscarriage was performed using the one-way analysis of variance (ANOVA) followed by the Post-Hoc test for multiple comparisons with the additional Kruskal-Wallis (KW) test and reported it in parameters with unequal distribution.

KW test was also used when assessing the differences between groups in percentage of change in ET between cycles for each group. Post-hoc Scheffe test was used to identify the coupled groups differences for equal variance such as differences between cycles of endometrial thickness (mm). Post hoc Bonferroni test was performed using the Non-parametric approach for assessment of change in ET (mm and %) between the study groups.

Changes in rates of ET thinning below the cutoffs of 6,7,8 mm between cycles in each group was tested by the McNemar Test.

The logistic regression univariate and multivariate models were applied to assess the effect of several variables on a dichotomous dependent variable. All tests applied were two-tailed, with a *p*-value of *P* < 0.05 considered statistically significant.

## Results

### Study population and miscarriage cycle data

Between 2006 and 2017 there were 327 couples at our IVF unit, which conceived and had first trimester miscarriage with at least one subsequent embryo transfer cycle in our unit. Ninety-six couples were excluded due to missing data regarding the miscarriage cycle or the subsequent cycle. The data required for the study was available for 231 couples. Eighty couples were conservatively managed, 67 were treated with Misoprostol and 84 underwent D&C. Allocation to one of the three management groups was determined according to the initial treatment approach chosen by the patients. Of the patients who received Misoprostol treatment, D&C was later performed in six patients due to incomplete expulsion of conception products and among the patients who chose expectant management, D&C was later performed in two patients due to incomplete expulsion of conception products. These patients were excluded from the analysis. Accordingly, in our analysis the conservative management group finally included 78 patients, the Misoprostol group included 61 patients and the D&C group included 84 patients.

D&C procedure data were available for 72.4% (63/87) of women. An attending performed the D&C in 85.7% (54/63) of women and US was used only in a few procedures (4/63). Vacuum curettage was performed in nearly all cases (93.6% (59/63)), followed by a sharp curettage in all cases. The ET in the following IVF cycle of patients after D&C by an attending physician (mean ET of 9.3 mm with an ET decrease of 1.1 mm between miscarriage and subsequent IVF cycles and only 3 out 54 patients with an endometrium thinner than 6 mm) or by a resident (mean ET of 8.4 mm with an ET decrease of 1.0 mm between miscarriage and subsequent IVF cycles and only 1 out of 9 patients with an endometrium thinner than 6 mm) was not significantly different.

The characteristics of the patients (Table 1) did not significantly differ between the groups, except for the rate of past deliveries (assessed by the Kruskal-Wallis test) which was lower in the conservative management compared to the D&C group. The groups did not differ with regard to the use of any medication, previous gynecologic operations, anatomical uterine abnormalities, previous ectopic pregnancy, rate of patients with previous miscarriages and previous D&C (the remaining cases were managed by expectant management or by Misoprostol), ovulation induction protocol (agonist or antagonist) and cause of infertility. The most prevalent causes of infertility were male factor (total 39.6%) and unexplained infertility (27.3%). Ovulation-related or mechanical factor were the cause of infertility in 22% of patients, and preimplantation genetic diagnosis (PGD) procedure was indicated in about 4% of them.

**Table 1:** Characteristics of the patients and the IVF cycle that gave rise to miscarriage conception in the different management groups

Parameter	Conservative management	Misoprostol	D&C	***P*** value
**N of patients**	78	61	84	
**Age (years)**	34.6 ± 6.6(35(29–41))	37.1 ± 5.0(39(33–41))	35.7 ± 5.6(37(31–40))	0.06
**BMI**	25.8 ± 2.8(25(23–28))	24.3 ± 6.1(23(19–30))	26.7 ± 7.8(24(21–30))	0.61
**Obstetric background**				
**Gravidity**	1.3 ± 1.5(1(0–2))	1.8 ± 2.2(1(0–3))	1.8 ± 1.8(2(2–3))	0.16
**Past deliveries**	28/72 (38.9%)	27/59 (45.8%)	49/81 (59.3%)	**0.04**
**Prior miscarriages**	0.8 ± 1.2(0(0–1))	1.1 ± 1.8(1(0–2))	0.9 ± 1.4(0(0–1))	0.41
**Patients with a previous D&C**	17.9%	25.5%	19.7%	0.83
**Infertility (years)**	4.1 ± 4.8(3(1–5))	2.6 ± 1.8(2(1–4))	4.2 ± 3.3(3(2–6))	0.08
**Day 3 FSH levels**^**a**^ **(mIU/ml)**	7.7 ± 6.9(6(5–9))	8.4 ± 7.5(7(6–9))	6.9 ± 3.1(7(5–8))	0.41
**Miscarriage cycle**				
**Peak E2 levels (Fresh cycles, pmol/l)**	6146.2 ± 3939.9(5477(2896–8593))	5910.9 ± 3464.5(4942(3928–7866)	6267.3.5 ± 4273.0(5654(3547–8104))	0.53
**Endometrium (mm) at hCG/LH/P**	9.9 ± 1.8(10(9–12))	10.1 ± 2.0(10(9–11))	10.1 ± 2.1(10(9–12))	0.81
**> 1 embryonal sac**	8(10.5%)	3(4.9%)	7(7.7%)	0.48
**Week of miscarriage**	6.7 ± 1.3(6(6–7))	7.7 ± 1.3(7(7–9))	8.6 ± 1.6(8(8–10))	**< 0.001**^**b**^
**Hysteroscopy for retained placenta**	3(4.2%)	1(1.8%)	0	0.13

Data presented as mean ± SD (Median (IQR)) or n (%)

Statistical analysis was performed using the Pearson Chi-Square test or Fisher’s Exact Test as indicated for categorical data and ANOVA or Kruskal-Wallis test (for variables with unequal distribution) for continuous parameters

*Note: BMI* Body mass index, *D&C* Dilation and curettage, *FSH* Follicular stimulating hormone, *hCG/LH/P* Day of human chorionic gonadotropin administration in fresh IVF cycles, luteinizing hormone surge or progesterone administration in frozen-thawed cycles

^a^Baseline FSH levels measured in hormone panel test at day three of menstrual period (prior to initiation of hormonal therapy)

^b^Post hoc Dunnett T3 test showed significant difference between all the study groups

A significant difference was noted in the gestational week at miscarriage diagnosis (*p* = 0.04) between all three management groups (by post hoc Dunnett test), ranging from 6.7 ± 1.3 weeks in the conservative management group to 8.6 ± 1.6 weeks in the D&C treatment group (Table 1).

The three management groups had similar proportions of fresh or frozen-thawed embryo transfers in the subsequent cycle, as well as similar time interval between cycles, peak Estradiol levels and embryo quality (presented as mean rates of embryos graded A-C) (Table 2).

**Table 2:** Basic parameters and endometrium thickness (ET) in the cycle consecutive to the miscarriage cycle of the different management groups

Parameter	Conservative management	Misoprostol	D&C	***P*** value
**N of patients**	78	61	84	
**Time from miscarriage cycle (months)**	5.4 ± 3.5 (4(3–6))	5.1 ± 2.4(5(3–6))	5.9 ± 2.9(5(4–7))	0.43
**Fresh consecutive cycle**	28/78(35.9%)	29/61(47.5%)	40/84(47.6%)	0.24
**Frozen-thawed consecutive cycle**	50/78(64.1%)	32/61(52.5%)	44/84(52.4%)	0.24
**Peak Estradiol levels (fresh cycle; pmol/l)**	4527.7 ± 2706.9(4268(3047–6472))	5164.9 ± 3639.4(4821(3528–6599)	4805.7 ± 2706.1(3961(2042–5623))	0.56
**Grade A embryos (%)**	27.1 + 37.7	28.0 + 36.2	31.3 + 36.1	0.91
**Grade B embryos (%)**	47.6 + 43.3	58.0 + 40.6	45.2 + 38.9	0.70
**Grade C embryos (%)**	6.3 + 18.8	1.3 + 3.7	10.1 + 22.8	0.21
**N of transferred embryos**	1.6 ± 0.8(2(1–2))	1.7 ± 0.8(2(1–2))	2.0 ± 1.0(2(1–3))	0.41
**ET (mm) at day of hCG/LH/P**	9.9 ± 2.1(10(8–11))	10.3 ± 2.3(10(9–11))	9.1 ± 2.4(10(9–11))	**0.005**^**b**^
**Comparison of ET between miscarriage cycle and consecutive cycle**^**a**^				
**Difference at day of hCG/LH/P (mm)**	0.0 ± 1.7(0(−1–1))	0.2 ± 2.2(0(−1–1))	−1.0 ± 2.4(−1(−2–0))	**0.002**^**b**^
**% of change at day of hCG/LH/P**	−0.9 ± 17.4(1(− 8–10))	3.5 ± 21.7(0(−8–10))	−8.2 ± 22.3(−8(−25–3))	**0.001**^**b**^
**Decrease of > 2 mm in ET at day of hCG/LH/P**	11/78(14.1%) ^C^	4/61(6.6%) ^C^	25/84(29.8%)	**< 0.001**
**Reproductive outcomes**				
**Implantation rate**	32/105 (30.5%)^C^	18/102 (17.7%)	16/144 (11.1%)	**< 0.001**
**Clinical pregnancy rate**	30/78 (38.5%)^C^	17/61 (27.9%)	14/84 (16.7%)	**0.008**
**Miscarriage rate**	12/30 (40.0%)	7/17 (41.2%)	5/14 (35.7%)	0.947
**Live birth rate**	18/78 (23.1%)	10/61 (16.4%)	9/84 (10.7%)	0.107

Data presented as mean ± SD (Median (IQR)) or n/N(%)

*Note: D&C* Dilation and curettage, *ET* Endometrial thickness, *hCG/LH/P* Day of human chorionic gonadotropin administration in fresh IVF cycles, luteinizing hormone surge or progesterone administration in frozen-thawed cycles

^a^Evaluated by one-way ANOVA and Kruskal-Wallis Test, showing significant difference in post hoc test between the conservative management and the D&C groups

^b^Evaluated by one-way ANOVA and Kruskal-Wallis Test, showing significant difference in post hoc test between both conservative and Misoprostol and the D&C group

^C^Evaluated by post hoc Bonferroni test, showing significant difference compared to the D&C groups

### The effect of miscarriage management on endometrial thickness in the subsequent cycle

Analysis of ET at miscarriage and subsequent cycles, at the time of hCG administration in fresh cycles or LH surge /progesterone administration in frozen-thawed cycles (hCG/LH/P), revealed significant differences between the miscarriage management approaches (Table 2). D&C was associated with a greater average reduction in ET per patient at hCG/LH/P day in the subsequent cycle compared to conservative management and Misoprostol (− 1.0 mm vs. 0.0 mm and 0.2 mm, respectively; *p* = 0.001). When analyzing the overall mean difference in ET in the miscarriage and the subsequent cycle for each group separately, there was a significant reduction in ET in the D&C group (1.0 mm decrease in ET, *p* = 0.002) while the mean ET was not significantly altered in both conservative management (*p* = 0.99) and Misoprostol (*p* = 0.60) groups.

In accordance with this finding, the rates of a significant ET decrease (more than 2 mm) at hCG/LH/P day between the cycles was significantly higher following D&C compared to the conservative and Misoprostol methods (29.8% vs. 14.1 and 6.6%, respectively; *p* < 0.001, assessed by post hoc Bonferroni test. Furthermore, tested by the McNemar test, management by D&C was the only approach associated with a significant increase in the rates of ET thinning under 7 and 8 mm in the following cycle (*p* = 0.006 and 0.035; respectively), while an increase in the prevalence of patients with thinning of the endometrium to below 7 and 8 mm was not demonstrated following conservative management or Misoprostol treatment. ET thinning below 6 mm, though showing similar trend in the D&C group, occurred in only few cases and did not reach statistical significance.

Univariate analyses, performed to examine the association between parameters of the miscarriage cycle and ET above 8 mm in the subsequent cycle, identified these factors: previous delivery, miscarriage management approach and ET at hCG/LH/P in the miscarriage cycle, both as a continuous parameter and as a categorical parameter (> 8 mm). In a multivariable logistic regression analysis, all of these 3 parameters were independently associated with endometrium thickness above 8 mm at hCG/LH/P in the following cycle (Table 3).

**Table 3:** Logistic regression analysis of parameters associated with endometrial thickness above 8 mm in the cycle subsequent to the miscarriage cycle

Parameter	OR	Lower 95% CI	Upper 95% CI	***P*** value
**Miscarriage management**				0.02
**D&C**	Referent			
**Conservative**	2.68	1.10	6.53	0.03
**Misoprostol**	3.44	1.28	9.25	0.01
**Past delivery**	2.40	1.08	5.32	0.03
**ET at day of hCG/LH/P in the miscarriage cycle**	9.38	4.11	21.39	< 0.001

*Note: D&C* Dilation and curettage, *ET* Endometrial thickness, *hCG/LH/P* Day of human chorionic gonadotropin administration in fresh IVF cycles, luteinizing hormone surge or progesterone administration in frozen-thawed cycles

To analyze possible biases that may affect ET (as a continuous parameter) at the subsequent cycle, the potential correlation between various patients’ parameters (basic characteristics including age, previous gynecologic operations, anatomical uterine abnormalities, previous Cesarean sections (CS), D&C or Misoprostol treatment, week of gestation, IVF protocol and fresh or frozen embryo transfer) and ET was evaluated. There was no correlation between any of the parameters and ET, including patients’ age and week of miscarriage. Linear modeling (ANCOVA) demonstrated that the only parameters significantly associated with ET at the subsequent cycle were the miscarriage management approach and ET at hCG/progesterone administration day in the miscarriage cycle (*p* < 0.05 for both).

### The effect of miscarriage management on subsequent cycle outcomes

Reproductive outcomes were analyzed in the three study groups to complement the ET analysis (Table 2). Implantation rate was significantly lower in the subsequent IVF cycle following D&C compared to conservative and Misoprostol management groups (11.1% vs. 30.5 and 17.7%, respectively; *p* < 0.001).

Additionally, clinical pregnancy rates were also significantly lower in the subsequent IVF cycle following D&C compared to conservative and Misoprostol managements (16.7% vs. 38.5 and 27.9%, respectively, *p* = 0.008). The main difference for both parameters was derived from the difference between the conservative management and the D&C group according to the post hoc analysis (post hoc Bonferroni test).

Although once a successful implantation occurred, there was no difference in miscarriage rates between the groups (*p* = 0.95). There was a trend towards a lower live birth rate in the D&C group compared with the conservative and Misoprostol management groups, though the difference did not reach statistical significance (10.7% vs. 23.1% and 16.4, respectively, *p* = 0.11).

A logistic regression analysis was performed for the prediction of clinical pregnancy in the cycle subsequent to the miscarriage cycle. Both univariate and multivariate analyses found that the only significant independent parameters associated with clinical pregnancy were the mother’s age at the following cycle and the miscarriage management approach (Conservative and Misoprostol compared to D&C) (Table 4).

**Table 4:** Logistic regression analysis for prediction of clinical pregnancy in an In-vitro fertilization cycle following a miscarriage cycle

Parameter	OR	Lower 95% CI	Upper 95% CI	***P*** value
**Miscarriage management**				0.022
**D&C**	Referent			
**Conservative**	2.68	1.34	6.10	0.01
**Misoprostol**	2.28	0.99	5.23	0.05
**Mother’s age**	0.91	0.86	0.96	< 0.001

*Note: D&C* Dilation and curettage

We further performed an additional analysis that included only patients in which the subsequent IVF cycle was of the same type (fresh or frozen-thawed) as the miscarriage cycle (Suppl. Table 1**)**. In line with the analysis of the entire study population above (Tables 1 & 2), we observed significant differences in the average ET at the day of hCG/LH/P administration between the miscarriage management approaches. D&C was associated with a greater average reduction in ET per patient at hCG/LH/P day in the subsequent cycle compared to conservative management and Misoprostol (− 0.9 mm vs. 0.0 mm and 0.5 mm, respectively; *p* = 0.004). Furthermore, management by D&C was the only approach associated with increased rates of ET thinning under 7 and 8 mm in the following cycle (*p* = 0.008 and 0.035; respectively).

Implantation and clinical pregnancy were significantly lower in the subsequent IVF cycle following D&C compared to conservative and Misoprostol management groups (Suppl. Table 1).

When comparison between the three management groups was performed separately for fresh and frozen-thawed cycles, similar significant results were demonstrated for most parameters. The peak E2 levels in the fresh cycles were similar in the three sub-groups. A significant (*p* < 0.05) decline in ET following D&C compared to similar ET after conservative management and Misoprostol was observed in both types of transfers. The difference in rates of a significant ET decrease (more than 2 mm) following D&C compared with the two other approaches was statistically significant (*p* < 0.05 for both types of cycles). In frozen-thawed cycles, clinical pregnancy rates were lowest in the D&C and highest in the conservative management group (*p* < 0.05). Although not significant, a similar trend was documented in the fresh transfer group (data available upon request).

## Discussion

In this study, we analyzed the effect of three miscarriage management approaches following an IVF cycle – expectant management (conservative), medical treatment (Misoprostol) and D&C, on endometrial thickness, implantation rates and pregnancy outcomes in the subsequent IVF attempt. To the best of our knowledge, this is the first study that compares ET at miscarriage IVF cycles with ET at subsequent IVF cycles with regard to the three miscarriage management approaches.

Our major finding was endometrial thinning in the subsequent IVF cycle following D&C compared with the other miscarriage treatment approaches. A statistically significant thinning of average ET was observed after D&C in comparison to the other management methods. This was only observed in the D&C group by a significant increase in the prevalence of ET thinning by more than 2 mm, and in the rate of patients who had ET decrease below 7 and 8 mm in the subsequent cycle.

Endometrial thinning after repeated D&C was seen in several previous studies [19–21]. Shufaro et al. (2008) reported on a spectrum of post-curettage endometrial injuries ranging from a thin and unresponsive endometrium to Asherman syndrome [22]. Nevertheless, Moon et al., suggested that the decrease in ET after D&C is transient, lasts no longer than 6 months and have little or no clinical meaning [23]. A previous study by Tamir et al. examined 41 patients treated with D&C and 32 patients treated with Misoprostol for spontaneous miscarriage following IVF cycles. They also showed a trend towards thinning of the endometrium after D&C, though not significant (10.4 mm before vs. 9.4 mm after D&C; *p* = 0.06). However, when comparing the two miscarriage management approaches, they reported a non-significant difference regarding ET on hCG administration day and similar pregnancy rates in subsequent cycles [24].

Our data suggested similar ET in subsequent IVF cycles after expectant management in comparison to medical treatment. Moreover, while following D&C, the ET was significantly thinner at the time of hCG/LH/progesterone administration in the subsequent IVF cycle in comparison to the miscarriage IVF cycle, the ET was not significantly altered after expectant and Misoprostol managements. It may be speculated that D&C may be associated with a deleterious effect on stem cells in the basilar layer of the endometrium, resulting in a potential adverse effect on endometrial regeneration [19]. In contrast, both Misoprostol and expectant management are not associated with surgical trauma to the endometrium and give rise to expulsion of conception by other mechanisms. Misoprostol softens the cervix, primarily through an inflammatory response that leads to collagen degradation [25] and induces uterine contractions [26]. As for expectant management, the mechanism of miscarriage includes entry of maternal blood into the intervillous space that disrupts the maternal–embryonal interface [27].

There is inconsistent data in the literature regarding the optimal ET for embryonic implantation and successful pregnancy outcome after IVF. The lowest thickness under which poorer implantation rate has been reported is variable in different studies ranging from 6 mm to 10 mm [3–14]. The different methodologies in the reported studies such as the cycle day at which ET was measured and diverse measurement settings probably contribute to the literature inconsistency. A recent comprehensive study that analyzed 40,000 embryo transfer cycles suggested a decline in clinical pregnancy and live birth rates at ET below 8 mm in fresh embryo transfers and below 7 mm in frozen-thawed embryo transfers [28]. It has been suggested that the decrease in pregnancy outcomes is related to increased exposure to higher oxygen concentrations in the endometrial basal layer when the endometrium is thinner, which might be detrimental for embryo implantation [29].

In line with the literature, we showed a significant reduction in implantation and clinical pregnancy rates in the D&C group, where the average ET was thinner, and the ET decrease below 7 and 8 mm in the subsequent cycle was significant. Although these findings were primarily due to the difference between the conservative and the D&C groups, whereas the Misoprostol showed a lesser advantage in implantation and pregnancy rates over the D&C group, our logistic regression model revealed that both conservative management and Misoprostol treatment were positively associated with clinical pregnancy at the subsequent cycle compared to D&C.

Our study was based on retrospective analysis of all patients that had a miscarriage after IVF treatment in our unit between 2006 and 2017. In an attempt to overcome, at least in part, the limitations of a retrospective study, we analyzed the ET before and after intervention, in the subsequent IVF cycle, per each patient to reduce interpatient variability that could mask the results. Except for previous deliveries and miscarriage week (which were highest in the D&C group), there were no differences between the groups regarding patients’ characteristics, gynecological history, cause of infertility and level of estradiol at hCG administration day. The differences between the study groups in the parameters of the miscarriage cycles regarding the number of previous deliveries and gestational age at miscarriage are limitation of the study. Nevertheless, linear and logistic regression analyses did not show that gestational week at miscarriage was correlated with ET, ET decrease below 8 mm or clinical pregnancy rates at the subsequent IVF cycle. Still, the gestational week at diagnosis could have an effect on the selection of the management modality by the patients. An additional limitation was the lack of data regarding the size of the gestational sac, the presence of an embryo and embryonal heartbeat loss in the miscarriage pregnancy. In contrast to a previous study that suggested a decrease in ET as a function of age [30]. A logistic regression analysis in our study did not find a correlation between patient’s age and ET, though the multivariate analysis for clinical pregnancy did show a positive association with younger age. Nevertheless, the patients in the D&C group were not older compared to the other study groups.

In conclusion, the results of the study suggest that D&C for miscarriage is associated with decreased ET and higher rates of thin endometrium below 7 and 8 mm in subsequent IVF cycles compared with expectant management and Misoprostol treatment. In addition, the implantation and clinical pregnancy rates of subsequent IVF cycles were significantly lower after D&C, compared to conservative and medical managements of miscarriage Our data may assist in counseling patients confronted with making the choice of management of a miscarriage after IVF as well as after a spontaneous pregnancy.

## Supplementary Information

**Additional file 1: Table S1.** Basic parameters, endometrium thickness (ET) and reproductive outcomes in the different management groups of consecutive similar type cycle to the miscarriage cycle.

## Data Availability

The datasets used and/or analyzed during the current study are available from the corresponding author on reasonable request*.*
