# Supplementary material for: Endometrial thickness following early miscarriage in IVF patients – is there a preferred management approach?
Source: Reprod Biol Endocrinol. 2021 Jun 22;19:93. doi: 10.1186/s12958-021-00780-7 (PMC8218428; doi:10.1186/s12958-021-00780-7)
Supplement: Supplementary file 1 — Additional file 1: Table S1. Basic parameters, endometrium thickness (ET) and reproductive outcomes in the different management groups of consecutive similar type cycle to the miscarriage cycle. [file 12958_2021_780_MOESM1_ESM.docx]

| **Suppl. Table 1.** Basic parameters, endometrium thickness (ET) and reproductive outcomes in the different management groups of consecutive **similar** type cycle to the miscarriage cycle | | | | | |  |
| --- | --- | --- | --- | --- | --- | --- |
| **P value** | | **D&C** | **Misoprostol** | **Conservative management** | **Parameter** | |
|  | | 59 | 41 | 56 | **N of patients** | |
| 0.023 | | 35.7±5.6(36(32-40)) | 37.1±5.0(39(35-42)) | 34.6±6.6(36(30-41)) | **Age (years)** | |
| 0.36 | | 7.2±3.9(6(4-9)) | 6.8±3.3(6(4-9)) | 8.1±4.7(8(4-11)) | **Time interval from miscarriage cycle (months)** | |
| 0.77 | | 6.1±2.7 (6(4-8)) | 5.6±2.8 (5(4-7)) | 5.9±2.4 (6(4-7)) | **ET (mm) at day 3** | |
| **<0.001** | | 29/59(49.2%) | 22/41 (53.7%) | 14/56 (25.0%) | **Fresh consecutive cycle** | |
| 0.62 | | 5383.3±4087.4(4082(2769-7249)) | 5488.0±4384.4(3909(3469-6116)) | 4156.1±2596.8(3283(1794-6558)) | **Peak Estradiol levels (fresh cycle; pmol/l)** | |
| 0.64 | | 2.1±1.1(2(1-3)) | 1.9±0.9(2(1-2)) | 1.9±1.0(2(1-2)) | **N of transferred embryos** | |
| **0.043** | | 9.3±2.5 (9(8-10)) | 10.5±2.5 (11(9-12)) | 9.9±2.3 (10(8-11))) | **ET (mm) at day of hCG/LH/P** | |
|  | |  |  |  | **Comparison of ET between miscarriage cycle and consecutive cycle** | |
| 0.06 | | 0.1±3.1 (0(-3-2)) | -0.3±2.2(0(-1-1)) | 1.2±2.4 (1(-1-3)) | **Difference at day 3 (mm)** |  |
| **0.004** | | -0.9±2.3 (-1(-2-0)) | 0.5±2.2 (1(-1-2)) | 0.0±1.9 (0(-1-1)) | **Difference at day of hCG/LH/P administration (mm)** |  |
| **0.003** | | 17/59(28.8%) | 3/41(7.3%) | 5/56(8.9%) | **Decrease of > 2 mm in ET at day of hCG/LH/P** |  |
|  | |  |  |  | **Reproductive outcomes** |  |
| **<0.001** | | 11/97 (10.7%) | 10/73 (13.7%) | 27/79 (34.2%) | **Implantation rate** |  |
| **0.007** | | 11/59 (18.6%) | 10/40 (25.0%) | 25/56 (44.7%) | **Clinical pregnancy rate** |  |
| 0.19 | | 5/11 (45.5%) | 6/10 (60.0%) | 7/25 (28.0%) | **Miscarriage rate** |  |
| **0.011** | | 6/59 (10.2%) | 4/40 (10.0%) | 18/56 (32.1%) | **Live birth rate** |  |

Data presented as mean± SD (median(IQR)) or n/N(%).

*Note:* D&C, dilation and curettage; ET, endometrial thickness; hCG/LH/P, day of human chorionic gonadotropin administration in fresh IVF cycles, luteinizing hormone surge or progesterone administration in frozen-thawed cycles.
